# Development and Characterization of Antioxidant and Antibacterial Films Based on Potato Starch Incorporating *Viola odorata* Extract to Improve the Oxidative and Microbiological Quality of Chicken Fillets during Refrigerated Storage

**DOI:** 10.3390/foods12152955

**Published:** 2023-08-04

**Authors:** Ali Nikmanesh, Homa Baghaei, Abdorreza Mohammadi Nafchi

**Affiliations:** 1 Department of Food Science and Technology, Damghan Branch, Islamic Azad University, Damghan, Iran; ali.nikmanesh@yahoo.com; 2 Food Technology Division, School of Industrial Technology, Universiti Sains Malaysia, Penang 11800, Malaysia; 3 Green Biopolymer, Coatings & Packaging Cluster, School of Industrial Technology, Universiti Sains Malaysia, Penang 11800, Malaysia

**Keywords:** active food packaging, biopolymer, *Viola odorata*, natural preservative

## Abstract

In this research, the antioxidant and antibacterial activities of active films based on potato starch containing *Viola odorata* extract (VOE) were investigated both in vitro and in chicken fillets. The VOE was added to the starch film formulation at 0, 1, 2, and 3% (*w*/*v*). The results showed that by increasing the extract level, the total phenol content and antioxidant and antibacterial activity of the films against *Escherichia coli, Staphylococcus aureus*, and *Salmonella typhimurium* improved remarkably. The results of the meat tests indicated the significant antioxidant and antimicrobial activity of active films containing different levels of VOE in chicken fillets, and a direct relationship was observed between the concentration of the extract and the functional activity of the films, so with the increase in the concentration of the extract in the films, the rate of lipid oxidation and growth of microorganisms in the chicken fillets decreased significantly during the storage period, and less volatile nitrogen bases, metmyoglobin, and oxidation products were produced in the fillets. In general, the results of this research demonstrated that an active film based on potato starch containing VOE (especially 2 and 3% levels) has the ability to extend the oxidative and microbiological shelf life of chicken fillets during cold storage for at least eight days.

## 1. Introduction

Meat and meat products, which are good sources of nutrients, especially proteins, are among the most consumed foods in the human diet. These products are highly perishable and spoil quickly, even in cold conditions [1]. The oxidation of fats is one of the reasons for the decline in the quality of meats over time, which causes an unpleasant taste and smell in the product and reduces the nutritional value of food. Microbial growth is also one of the major causes of spoilage in meat products, which is dangerous for humans and reduces the shelf life of products [2]. The application of active packaging film is one of the effective ways to increase the shelf life of food products and improve their safety and stability [3]. The release of bioactive compounds from packaging materials into food products can prevent the oxidation of fats and the growth of microorganisms [4].

The packaging used for food products is based on synthetic materials that are nonrenewable and nonbiodegradable and have high resistance and good barrier properties. Still, they produce a huge amount of waste material and create environmental problems [5]. However, the use of packaging is one of the necessities in the food industry. Packaging plays an essential and important role in controlling the reactions between food and its surrounding environment, protecting food against environmental conditions, and preserving product quality during transportation and storage [6]. In order to solve the problems caused by the use of synthetic packaging, attention has been paid to the use of biodegradable and renewable materials, such as carboxymethyl cellulose, proteins, chitosan, carbohydrates, fatty acids, etc., for the development of food packaging [7,8]. To develop active films, active additives are integrated into the formulation of biopolymer films, which in addition to the ability to increase the shelf life and safety of packaged food products also affect the physical and chemical properties and performance of packaging. The natural agents can also play the roles of blocking UV light, lubricants, plasticizers, etc. [9,10]. Plants are abundantly distributed in nature, and many of their different parts, such as flowers, leaves, roots, and seeds, often have various bioactive compounds and demonstrated functional and biological properties and can be used in biopolymer films [11]. By combining biopolymers with natural bioactive compounds, active films with new and improved characteristics can be developed [12]. *Viola odorata* or sweet Banafsheh, is a member of the *Violaceae* family and has a long history in Iranian traditional medicine. It has been used to treat diabetes, migraine, anxiety, blood pressure, cancer, and as a sedative. The antimicrobial and antipyretic effects of this plant have also been shown [13,14]. *Viola odorata* is a good source of various bioactive compounds such as flavonoids, triterpenoids, alkaloids, phenolic acids, and cyclotides, and the antioxidant and antimicrobial activity of its extracts have been reported in previous studies [15,16].

In this research, a novel active film using potato starch as the base film polymer and *Viola odorata* extract as an active agent with antioxidant and antimicrobial activities was developed, and after examining the characteristics of the produced films, the possibility of using these active films to extend the shelf life and improve the quality characteristics of chicken breast fillets during the storage period in a refrigerator was investigated.

## 2. Materials and Methods

### 2.1. Materials

*Viola odorata* flowers were obtained from a local market in Babol (Mazandaran, Iran) and were approved at the Medicinal Plants Institute of Azad University (Damghan, Iran). Potato starch was obtained from SIM Supply Company (Penang, Malaysia), and microbial strains, including *Staphylococcus aureus*, *Salmonella typhimorium*, and *Escherichia coli*, were purchased from Iran’s Scientific and Industrial Research Organization. The culture media, including Plate Count Agar, Tryptone Soy Agar, Violet Red Bile Agar, and Baird-Parker Agar, and chemicals were purchased from Merck (Darmstadt, Germany).

### 2.2. Preparation of Viola odorata Extract (VOE)

*Viola odorata* flowers were dried in the shade and then turned into powder by an electric mill (Pars Khazar, Jajarm, Iran), and the resulting powder was passed through a sieve with a mesh of 250 µm. After that, 300 g of plant powder was added to 1 L of distilled water and mixed slowly on a magnetic stirrer for 24 h at room temperature. Then, the mixture of solvent and plant was filtered using Whatman filter paper (No. 1), the primary extract was transferred to a rotating evaporator, and its solvent was evaporated under vacuum at a temperature of 40 °C. The extraction yield of *Viola odorata* extract (VOE) was 17.9% [13].

The total phenol content (TPC), total flavonoid content (TFC), and total anthocyanin content (TAC) of the VOE were determined using the Folin–Ciocalteu standard method [17], aluminum chloride colorimetric method [18], and pH differential method [19], respectively. A 1, 1-diphenyl-2-picryl-hydrazyl (DPPH) radical scavenging assay was used to determine the antioxidant activity of the VOE [20]. The TPC, TFC, TAC, and antioxidant activity of the VOE were 53.41 mg GAE/g dried extract, 22.19 mg QE/g dried extract, 1.81 cyanidin-3-glycoside, and 83.79%.

### 2.3. Preparation of Potato Starch/VOE Active Films

To prepare the starch films, 4 g of potato starch was added to 100 mL of distilled water and stirred, and then the starch suspension was heated using a hot plate. After the starch suspension reached 40 °C, 25% (*w*/*v*) glycerol was added to it, and stirring was performed. Then, the resulting mixture was heated to 85 °C for 20–30 min to gelatinize the starch. After cooling the mixture to a temperature of less than 40 °C, different levels of VOE (1%, 2%, and 3% *w*/*v*) were added to the film solution and stirred well for 10–15 min using a magnetic stirrer. The resulting solutions were ultrasonicated for 10 min to remove air bubbles. After cooling the film solutions, 35 mL of each solution was poured into 140 mm diameter Petri dishes and placed at room temperature for two days to dry. After drying, the films were separated from the Petri dishes and kept in a desiccator until further use [21].

#### 2.3.1. Determination of Thickness and Mechanical Properties

The thickness of the films was measured at three points on each film using a QLR IP54 digital micrometer (Model QLR digit-IP54, Qinghai, China) with an accuracy of 0.001 mm.

The mechanical properties of the starch films were measured using a LLOYD texture analyzer (Model LRX, Lloyd Instruments, Hampshire, UK) and according to the ASTM D882-10 standard method. The determined mechanical parameters included the tensile strength (TS; MPa), Young’s modulus or elastic modulus (YM; MPa), and elongation at the break point (EAB; %). The films were cut into rectangular strips with a 100 mm length and 15 mm width and placed between the two jaws of the device. The initial jaw separation and the test speed were set to 50 mm and 0.5 mm/s, respectively. During the stretching time of the films, the force and distance were recorded until the breaking point, and each sample was tested at least three times.

#### 2.3.2. Determination of Barrier Properties

The water vapor permeability (WVP) of the films was measured using the ASTM E96-02 standard method. At first, 3 g of anhydrous calcium chloride was poured into small vials, and the surface of the glass was covered with film. After initial weighing, all the samples were simultaneously transferred to a desiccator containing magnesium nitrate (55% RH at room temperature). The changes in the weight of the vials over time were measured, and the slope of the resulting line was used for calculations. At first, the water vapor transmission rate (WVTR) was calculated, and then the WVP was determined using the following equations, where G is the weight change (g), t is time (min), A is the surface area of the vials (m^2^), P is the vapor pressure of pure water at 25 °C (Pa), R_1_ is the RH of desiccator (55%), R_2_ is the RH of a vial inside (0%), and X is the film thickness (m).

(1) $$\mathrm{WVT}=\frac{G}{t\times A}$$

(2) $$\mathrm{WVP}=\frac{WVT}{P(R1-R2)}\times X$$

The oxygen permeability (OP) of the starch films was measured using an Mocon Ox-Tran^®^ 2/20 apparatus (Modern Controls Inc., Minneapolis, USA) and according to the ASTM D3985-10 standard method. The surface area of the films exposed to nitrogen and oxygen was 50 cm^2^, and this test was performed at an RH of 75% and a temperature of 25 °C. The OP of the films was calculated by dividing the oxygen transmission by the partial oxygen pressure difference between the two sides of the film and multiplying it by the average thickness of the film and was reported as cc.µm/h.m^2^.kPa.

#### 2.3.3. Determination of Opacity and UV–Visible Transmission

In order to investigate the percentage of UV–visible transmission, the optical absorption of the films was read at 280 nm for UV light and at 600 nm for visible light [22]. To determine the opacity, first, the film was cut into rectangular strips of 40 mm length and 8 mm width, and then its absorbance was read using a spectrophotometer at 600 nm, and finally, the amount of opacity was obtained using the following equation [23]:

(3) $$\mathrm{Opacity}\;({\mathrm{mm}}^{-1})=\frac{A600}{Thickness(mm)}$$

#### 2.3.4. Determination of Total Phenol Content and Antioxidant Activity

The Folin–Ciocalteu standard method was used to determine the total phenol content (TPC) of the starch films. At first, 25 mg of each sample was dissolved in 3 mL of ethanol, and the resulting solution (0.3 mL) was transferred to a test tube, and 2.5 mL of Folin–Ciocalteu reagent (diluted tenfold with distilled water) and 2 mL of sodium carbonate (7.5% *w*/*v*) were added to it. The tubes were vortexed, covered with parafilm, and kept at a temperature of 50 °C for 5 min. Finally, the absorbance of the solutions was read using a spectrophotometer at 760 nm. Using the standard curve of gallic acid, the TPC values of the films were obtained and reported as milligrams of gallic acid equivalents per gram of film (mg GAE/g) [24].

A DPPH radical scavenging assay was used to determine the antioxidant activity of the films. At first, 25 mg of each film was dissolved in 3 mL of methanol, and then 2.8 mL of it was mixed with 0.2 mL of 1 mM DPPH methanolic solution. The resulting mixtures were vortexed at 2500 rpm for 1 min and kept in the dark for 1 h. The absorbance of the prepared samples was read at 517 nm against a methanol control. The DPPH radical scavenging percentage of film samples was obtained using the following equation [24]:

(4) $$\mathrm{DPPH}\;(\%)=\frac{A\;control-A\;sample}{A\;control}\times 100$$

#### 2.3.5. Determination of Antibacterial Activity

Determining the antibacterial activity of the films against *E. coli*, *S. aureus*, and *S. typhmurium* bacteria was achieved according to the agar disk diffusion method and determining the diameter of the growth inhibition zone around each disk. At first, 100 µL of bacteria suspension (containing approximately 5 logs CFU/mL bacteria) was spread evenly on a Tryptone Soy Agar (TSA) culture medium using a sterile swab. Films containing the extract with a diameter of 2.5 cm were prepared and placed on the inoculated culture medium, and then the plates were incubated for 24 h at a temperature of 37 °C. The diameter of the growth inhibition zone was reported as the antibacterial activity of the films [25].

### 2.4. Preparation of Chicken Fillets

The freshly slaughtered chicken was purchased from a local market (Semnan, Iran) and was quickly transferred to the laboratory in ice boxes. From the chicken breast meat, fillets with almost the same dimensions and weight (~50 g) were prepared manually, and after washing them with water, the fillets were placed on sterile plastic colanders. The prepared chicken fillets were packed in active films containing different levels of VOE and then placed in polyethylene bags and stored in a refrigerator (4 °C) for 12 days, and every 4 days, tests were performed on the fillet samples.

#### 2.4.1. Determination of pH and TVB-N

To measure the pH values of the meat samples using a pH meter (Jenway pH meter 350; Dunmow, Essex, UK), first, the device was calibrated by buffer solutions with different pH, and then 10 g of meat was poured into a container with 90 mL of distilled water and stirred with a glass rod. Finally, the pH value of the sample was read and recorded by the pH meter at room temperature [26].

To determine the total volatile basic nitrogen (TVB-N) values of the meat samples, 10 g of minced meat was mixed with 2 g of magnesium oxide and 300 mL of distilled water and poured into a Kjeldahl apparatus, and the device was heated. Then, the distillation continued for 45 min from the boiling time of the material inside the balloon by collecting about 100 mL of liquid, then inserting a 250 mL Erlenmeyer flask containing 25 mL of 2% boric acid solution (2 g of boric acid made up to volume with distilled water) and a few drops of 0.1% methyl red. Finally, the resulting solutions were titrated using sulfuric acid (0.1 N), and the TVB-N values of the fillets were calculated through the following equation and reported as mg N/100 g [27]:

TVB-N (mg/100 g) = Volume of sulfuric acid × 14(5)

#### 2.4.2. Determination of Fat Oxidation

To determine the peroxide index (POV) of the chicken fillets, 10 mg of minced meat was homogenized with 50 mL of methanol–chloroform solvent in a ratio of 1:2 (*v*/*v*) for 2 min. Then, it was filtered, and the solvent was removed. After that, the fat sample was mixed with 25 mL of chloroform–acetic acid solvent in a 2:3 (*v*/*v*) ratio, and 1 mL of saturated potassium iodide was added to the mixture. After being placed in the dark for 5 min, the mixture was mixed with 75 mL of distilled water and 0.5 mL of starch reagent and finally was titrated with 0.01 N sodium thiosulfate, and the POV of the fillets was calculated using the following equation, where S is the volume of used sodium thiosulfate (mL), N is the normality of the sodium thiosulfate solution, and W is the weight of the sample (kg) [28].

(6) $$\mathrm{Peroxide}\;\mathrm{value}\;(\mathrm{meq}/\mathrm{kg}\;\mathrm{sample})=\frac{S\times N}{W}\times 100$$

To measure the thiobarbituric acid index (TBA) of the fillets, the first 1 g of the sample was homogenized with 8 mL of trichloroacetic acid solution (5 g in 100 mL of water) and 5 mL of BHA solution (0.8 g in 100 mL of N-hexane). After centrifugation for 10 min at 3000 rpm and separating the upper phase, 2.5 mL of the lower phase was removed and homogenized with 1.5 mL of TBA solution (0.8 g in 100 mL of water). After heating at 75 °C for 30 min and then cooling, the absorbance of the samples was recorded at 532 nm, and the TBA value was obtained using the standard curve of 1,1,3,3-tetra ethoxy propane (0–0.8 µM). The results of the TBA index were reported as mg of malondialdehyde per kg of meat (mg MDA/kg meat) [29].

#### 2.4.3. Determination of Protein Oxidation

The carbonyl content of meat samples was measured to determine protein oxidation. First, 2.5 g of minced meat was homogenized with 8 M urea solution, and after adding 1 mL of 10% trichloroacetic acid solution to it, the mixture was centrifuged for 5 min at 5000× *g*. One mL of 2 M hydrochloric acid and an equal volume of 2% hydrochloric acid containing 0.2% *w*/*v* of dinitrophenylhydrazine (DNPH) were added separately to 100 µL of the sample. The samples were dissolved in a 20 mM phosphate buffer solution containing 1 mL of 6 M guanidine hydrochloric acid with a pH of 6.5. The resulting solution was stirred and centrifuged for 2 min at 4200× *g*, and finally, its absorbance was read at 280 nm. Bovine serum albumin was used as the standard, and the carbonyl content of the samples was reported as nano mol of DNPH per mg of protein (nmol DNPH/mg protein) [30].

#### 2.4.4. Determination of Metmyoglobin Content

One gram of the sample was weighed in a Falcon tube, and 5 mL of 6.8 phosphate buffer solution was added to it and then homogenized using an ice bath and with a homogenizer at 13,500 rpm for 10 s. After refrigeration for 1 h, it was centrifuged at 3500× *g* for 30 min. After that, the supernatant was passed through a Whatman filter (No. 42), and its optical absorbance was read at 525 nm, equivalent to the amount of metmyoglobin in the sample [31].

#### 2.4.5. Determination of Microbial Load

Different dilutions of each sample were prepared to count the microbial load of the chicken fillets, in such a way that 10 g of the sample was carefully weighed in a sterile container, and 90 mL of sterile distilled water was added to it, and the resulting mixture was left in a still place for 15 min to settle the coarse particles and then filtered using a Whatman filter (No. 42), and a dilution of 10^−1^ was obtained, and in the same way, other dilutions were also prepared.

Plate Count Agar (PCA) culture medium was used to count the total viable bacteria (TVBC), and the plates were incubated for 2 days at 37 °C. To count total coliforms, Violet Red Bile Agar (VRBA) culture medium was used, and the plates were incubated for 24 h at 37 °C for 24 h [32]. Baird-Parker Agar culture medium was used to count coagulase-positive staphylococci, and the plates were incubated for 48 h at 37 °C [33]. After the incubation period, the number of colonies was counted, and the results were reported as log CFU/g meat.

### 2.5. Statistical Analysis

Each test was repeated three times, and the mean of each parameter was analyzed by a one-way ANOVA using SPSS 22.0. The differences between samples were expressed using a Duncan’s multiple range test at a 95% probability level (*p* < 0.05).

## 3. Results and Discussion

### 3.1. Characteristics of the Active Film

#### 3.1.1. Thickness and Mechanical Properties

Thickness is one of the major parameters of packaging films and significantly affects the mechanical, barrier, structural, and thermal characteristics of films. The average thickness values of the produced films in the present research were in the range of 0.107–0.111 mm, and adding VOE and increasing its level did not significantly affect the thickness of the starch-based films (Table 1). Similarly, in some other research, the addition of plant extracts and essential oils did not have a significant effect on the thickness of biopolymer films [34,35].

Tensile strength (TS) is the maximum force that can be applied to films without damage or destruction. Elongation at the break point (EAB) is the maximum change in the length of films before they break. Young’s modulus (elastic modulus) is also one of the mechanical parameters related to the strength of a film structure [21]. In Table 1, the mechanical parameters of the active films are compared with each other. It shows that the control film had the highest TS (39.84 MPa) and YM (296.72 MPa), and adding 1% VOE decreased the TS and YM values to 38.52 MPa and 293.19 MPa, respectively; however, there was no statistically significant difference between this sample and the control film. With an increase in the VOE level, the TS and YM decreased and reached the lowest values in the film containing 3% VOE (36.67 MPa and 286.44 MPa, respectively).

Regarding EAB, the lowest value was observed in the control sample (11.09%), and with an increase in the VOE level, its value gradually increased, and the highest value was for the film containing 3% VOE (12.39%). Research has shown that phenolic compounds can act as a plasticizer and increase the flexibility of biopolymer films [36] because the phenolic compounds of extracts can lead to the easier movement of polymer chains through the weakening of intermolecular forces between macromolecules [37]. In the study of Nouri and Nafchi [38], in agreement with the results of the present study, adding betel extract decreased the TS and YM of sago-starch-based films and increased the EAB.

#### 3.1.2. Barrier Properties

**Figure 1:**
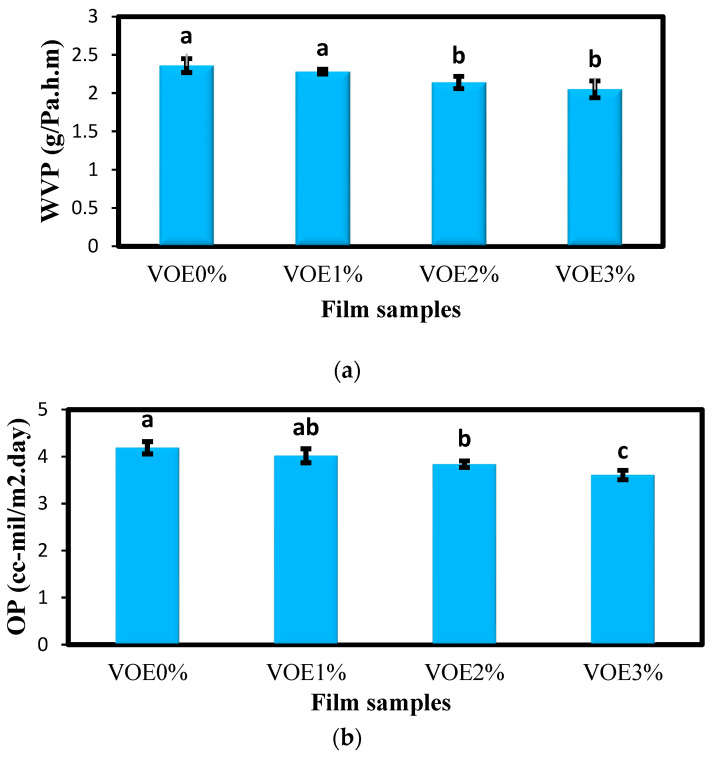
(**a**) Oxygen permeability (cc-mil/m^2^.day) and (**b**) water vapor permeability (10^−11^ g/Pa.h.m) of active films containing different levels of VOE. Bars represent the mean (*n* = 3) ± SD. Different letters on the bars indicate significant differences among samples at a 5% probability level. VOE: *Violata orodata* extract; OP: oxygen permeability.

Permeability to water vapor (WVP) is considered one of the major characteristics of a packaging film. Films with lower WVP have higher protective and barrier effects and better sewability [21]. The results of the investigation of the WVP of starch films (Figure 1a) showed that the highest value of WVP was for the control film (2.36 g/Pa.h.m), and the addition of 1% VOE led to a decrease in the WVP value to 2.28 g/Pa.h.m. By adding 2% and 3% VOE to the films, a significant decrease in WVP value was observed, and the WVP of these two active films was 2.14 and 2.05 g/Pa.h.m, respectively. The decrease in the WVP of biopolymer films due to the addition of red apple pomace and raspberry extracts was also reported by previous studies. The researchers attributed the decrease in WVP to the entry of bulky aromatic groups of phenolic compounds into the structure of biopolymer films, which reduced the films’ permeability to water vapor [39]. Riaz et al. [40] also stated that the cross-links between the phenolic compounds, glycerol, and polymer matrix could be the reason for the decrease in the WVP of the films due to the incorporation of phenol-rich extracts.

One of the important causes of food spoilage is the oxidation of fats, a destructive chemical reaction that occurs when food is exposed to oxygen. Therefore, suitable and desirable food packaging should have good barrier properties against oxygen in order to protect the packaged food product against the oxidation of fats [41]. The results of examining the oxygen permeability (OP) of the starch films (Figure 1b) showed that the control sample had the highest OP (4.19 cc.µm/h.m^2^.kPa). With an increase in the VOE level of the films, the OP decreased and reached the lowest value in the film containing 3% VOE (3.61 cc.µm/h.m^2^.kPa). In general, due to the addition of VOE, bulk hydroxyl groups enter the structure of biopolymer films, which can reduce the holes in the film structure and reduce the OP by reducing the passage of oxygen and other gases. Similarly, a decrease in the OP of starch-based films due to the incorporation of different levels of red cabbage extract was also reported in the research of Cheng et al. [42]. García et al. [35] also found that the addition of olive extract rich in polyphenols caused a slight decrease in the OP of corn-starch-based films.

#### 3.1.3. Opacity and Transmission of UV–Visible Light

Light is one of the driving factors in the oxidative spoilage of food products, so the films used for food packaging should prevent the passage of light as much as possible [43]. In Table 2, the opacity values and UV–visible light transmission percentage through the starch films are given, and it shows that the control film had the lowest opacity (0.347 mm^−1^) and the highest transmission of UV (44.61) and visible light (75.24%). Adding the VOE and increasing its level from 1% to 3% in the films significantly increases the opacity and UV–visible light transmission percentage (*p* < 0.05). The highest amount of opacity (1.014 mm^−1^) and the lowest amount of visible light transmission (17.87%) were related to the film containing 3% VOE. In the films containing 2% and 3% VOE, UV light transmission was not observed, which indicates the significant barrier of active films containing high levels of VOE against UV light. As the results showed, adding the VOE and increasing its level in the films increased the opacity, and this increase in opacity can increase the absorption of light by the films and reduce the percentage of light passing through them. The increase in the opacity of the films due to the addition of VOE is related to the presence of anthocyanin pigments in the flowers of this plant, such as quercetin-3-*O*-α-rhamnopyranosyl and cyaniding-3-(coumaroyl)-methylpentosyl-exosyl-5 exoside [44]. In agreement with these results, Cheng et al. [42] and Ali et al. [45] observed an increase in the opacity of starch-based films due to the incorporation of red cabbage extract and pomegranate peel rich in anthocyanins, respectively. Similarly, in another study, the addition of fruit extract led to an increase in opacity and a decrease in the percentage of UV light transmission in the starch films [46].

#### 3.1.4. Total Phenol Content and Antioxidant Activity

**Figure 2:**
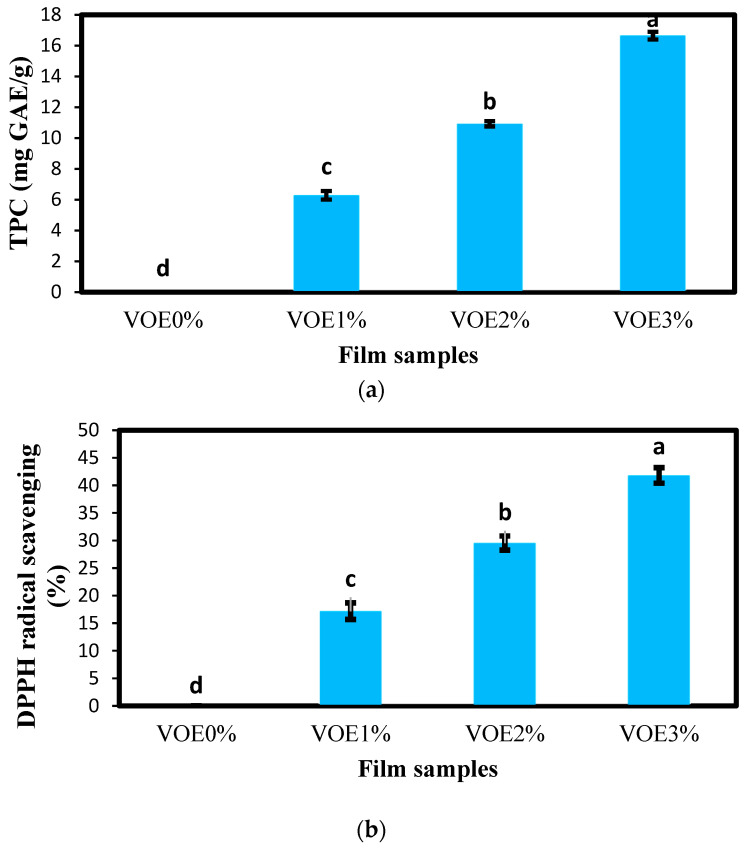
(**a**) Total phenol content (mg GAE/g) and (**b**) DPPH radical scavenging (%) of active films containing different levels of VOE. Bars represent the mean (*n* = 3) ± SD. Different letters on the bars indicate significant differences among samples at a 5% probability level. VOE: *Violata orodata* extract.

In this research, the total phenol content (TPC) of the films was tested using the Folin–Ciocalteu method. Their antioxidant activity was examined using a DPPH radical scavenging assay, and the results are presented in Figure 2. The control sample had no phenolic compounds and did not show antioxidant activity. As expected due to the presence of phenolic compounds in the VOE, by adding this extract and increasing its level from 1% to 3% in the films, the TPC and antioxidant activity of the films increased significantly (*p* < 0.05). The TPC values of the films containing 1%, 2%, and 3% VOE were 6.29, 10.93, and 16.65 mg GAE/g, respectively, and their DPPH radical scavenging percentages were 17.19%, 29.55%, and 41.82%, respectively. Antioxidant activity generally depends on the ability of antioxidants to donate hydrogen. Therefore, as the concentration of antioxidants increases, hydroxyl groups also increase, and with the increase in the ability of free radicals to donate hydrogen, the antioxidant activity also increases [47]. The VOE contains various bioactive compounds such as glycosides, saponins, alkaloids, methyl salicylate, phenolic acids, flavonoids, ascorbic acid, etc., indicating remarkable antioxidant activity [48]. The presence of high amounts of phenolic compounds in VOE was also found in the research of Jamshed et al. (2019). The good antioxidant activity of VOE has also been confirmed by Alipanah et al. [49] and Ibraheem et al. [48].

#### 3.1.5. Antibacterial Activity

The antibacterial activity of potato-starch-based films containing different levels of VOE against three strains of pathogenic bacteria, *E. coli*, *S. aureus*, and *S. typhimurium*, is shown in Table 3. The film without the extract (control) had no antibacterial activity, but by adding the VOE and increasing its level from 1% to 3% in the films, due to the increase in the content of bioactive compounds such as phenolic and flavonoid compounds, the antibacterial activity against pathogenic bacteria increased significantly (*p* < 0.05). The largest growth inhibition zones against *E. coli* (29.96 mm), *S. aureus* (34.10 mm), and *S. typhimorium* (31.50 mm) were observed in the film containing 3% VOE. Due to various bioactive compounds, plant extracts often show remarkable antimicrobial activity. Phenolic compounds attack the cell membrane and affect its permeability and cause the release of the main intracellular components such as sodium glutamate, ribose, etc., and destroy the functions of electron transfer, absorption of nutrients, ATPase enzymes, and DNA synthesis [50]. Ramezani et al. [51] also showed the significant antibacterial activity of VOE and attributed this activity to the presence of phenols, flavonoids, pectin polysaccharides, salicylic acid, and special proteins such as cyclotides. In other studies, the significant antibacterial activity of VOE against different pathogenic bacteria was also confirmed [52]. In most research, the antibacterial activity of plant extracts against Gram-positive bacteria has been higher than Gram-negative bacteria [53]. Researchers have stated that the higher resistance of Gram-negative bacteria is related to the structure of their cell walls because these bacteria have a thick layer and outer membrane of peptidoglycan, which shows a protective effect [54].

### 3.2. The Characteristic of Chicken Fillets

#### 3.2.1. PH and Total Volatile Basic Nitrogen (TVB-N)

**Figure 3:**
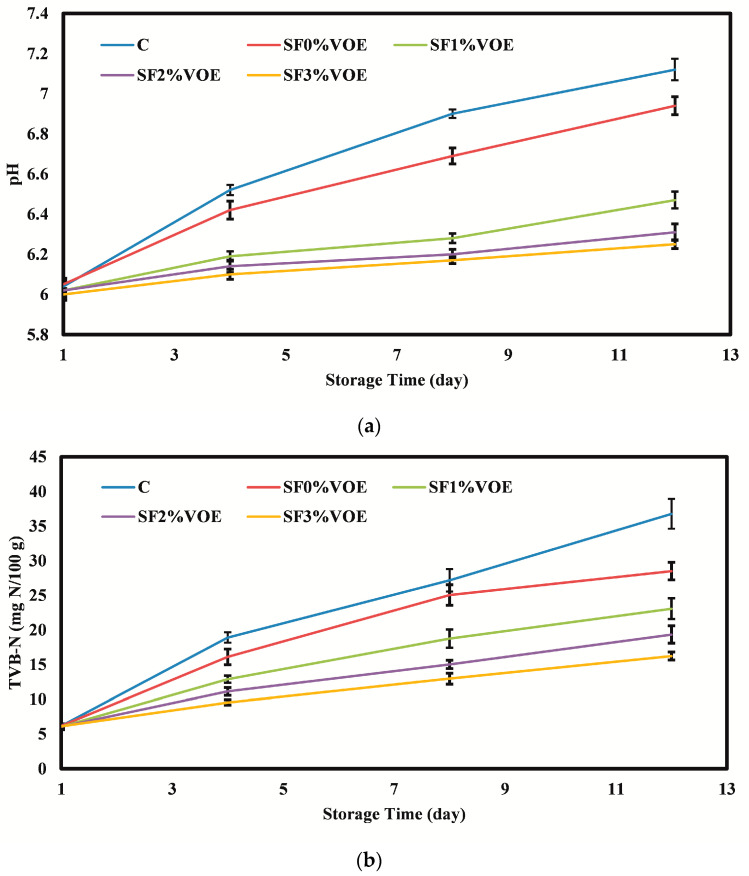
(**a**) Changes in the pH and (**b**) TVB-N values of chicken fillets during the cold storage period. C: control sample; VOE: *Violata orodata* extract; TVB-N: total volatile basic nitrogen.

The results of studying the pH values of chicken fillets are shown in Figure 3a. In the beginning, the pH values of the fillets were in the range of 6.00–6.04, and during cold storage, due to the activity of protease enzymes, as well as the activity of spoilage microorganisms and the breakdown of protein and the production of a higher amount of total nitrogenous bases [55], the pH of the samples increased significantly (*p* < 0.05) and reached its maximum values on the 12th day of storage (6.25–7.12). These results aligned with the results reported by other researchers [56]. On different days, the highest pH value was observed in the control sample, and with an increase in the level of VOE in the starch films, the pH changed significantly and decreased compared to the control (*p* < 0.05), and in all periods, the lowest pH values were found in the fillets packed in the film containing the highest level of VOE. Due to the presence of remarkable amounts of phenolic compounds, plant extracts and essential oils have antimicrobial activity and are able to protect meat samples against microorganism function and protease enzymes and prevent the breakdown of proteins and the production of amines [57]. Souza et al. [58] also achieved similar results in investigating the effects of active films containing ginger essential oil on the quality of chicken fillets.

The total volatile basic nitrogen (TVB-N) index is used to evaluate the volatile nitrogen bases (ammonia, trimethylamine, dimethylamine, etc.) of meat and meat products, which are caused by the activity of proteolytic enzymes, spoilage microorganisms, and the deamination of amino acids and nucleotides [59]. The results of evaluating the TVB-N values of chicken fillets (Figure 3b) showed that, initially, the TVB-N values were in the range of 6.11–6.18 mg N/100 g, and over time, due to the activity of internal proteolytic enzymes, as well as enzymes secreted by microorganisms, more volatile compounds were produced in the samples, and therefore during the cold storage period, a significant increase in the TVB-N content of the fillets was observed, and the highest values were on the 12th day of storage (16.22–36.77 mg N/100 g). With the increase in the level of VOE in the starch films due to the increase in antimicrobial activity, the production of volatile nitrogenous bases in the samples packed in the active films significantly decreased compared to the control sample (*p* < 0.05). The maximum acceptable amount for TVB-N in chicken meat is recommended to be 25 mg N/100 g [60]. The results of the present research indicate that the control sample and the sample packed in a film without the extract had values higher than 25 mg N/100 g from the 8th day of storage, and the other samples had less than 25 mg N/100 g until the 12th day of cold storage. Saleh et al. [56] agreed with these results and found that during cold storage, the TVB-N values of chicken samples increased; however, the use of olive leaf extract and increasing its level could significantly reduce the intensity of TVB-N production in the samples. Hematizad et al. [61] also reported the effect of active films containing *Zataria multiflora* essential oil in reducing the TVB-N of chicken breast meat.

#### 3.2.2. Oxidation of Fats

**Figure 4:**
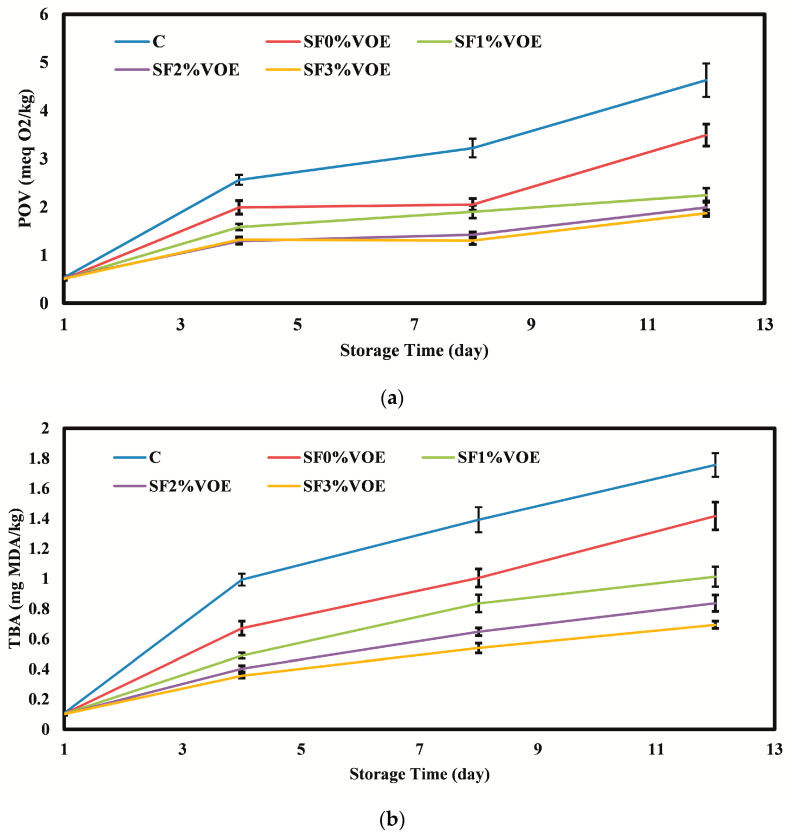
(**a**) Changes in the POV and (**b**) TBA index values of chicken fillets during the cold storage period. C: control sample; VOE: *Violata orodata* extract; POV: peroxide value; TBA: thiobarbituric acid.

The oxidation of fats is one of the destructive chemical reactions responsible for the deterioration of the quality of food products containing oil or fat during storage [62]. The peroxide index (POV), as one of the quality indicators, measures the concentration of hydroperoxides in foods, and therefore it presents the primary products resulting from the oxidation of fats. The thiobarbituric acid index (TBA) is also one of the most widely used oxidative indices to evaluate the rate of fat oxidation in food products. It is used to determine the secondary products resulting from the oxidation of fats, especially aldehydes [63]. The results of the investigation of the oxidation indices of the chicken fillets (Figure 4) showed that, initially, the POV and TBA values of the fillets were in the range of 0.50–0.53 meq/kg and 0.100–0.108 mg MDA/kg, respectively.

During cold storage, due to the breakdown of fats and the production of free fatty acids by internal lipases and lipase enzymes produced by microorganisms, the oxidation rate of fats increased. More hydroperoxides and malondialdehydes were also produced in the samples (*p* < 0.05). The highest values of POV (1.87–4.63 meq/kg) and TBA (0.693–1.756 mg MDA/kg) were observed on the 12th day of storage. On different cold storage days, the highest POV and TBA values were observed in the control sample, and the lowest values were for the fillets packed in the film containing 3% VOE. One of the reasons for the reduction in oxidation indices due to the use of active films is the reduction in the contact intensity of the meat samples with oxygen. Oxygen is an important factor in the initiation and development of fat oxidation. As reported in the first stage of the present study, the films containing VOE had a low permeability to oxygen. In general, edible coatings and films reduce the contact rate of food with oxygen by creating a protective layer against gases on the surface of foods, thereby significantly reducing the rate of fat oxidation [64]. On the other hand, plant extracts can donate hydrogen atoms from their structure to free radicals, thereby neutralizing and deactivating free radicals and reducing the rate of fat oxidation [65]. Phenols and flavonoids also have the ability to chelate the iron contained in the lipoxygenase enzyme and prevent the initiation of the oxidation reaction of fats [66]. Flavonoids, terpenoids, alkaloids, and phenolic acids have been isolated as important bioactive compounds of *Viola odorata* with good antioxidant activity [15]. The remarkable antioxidant activity of VOE was also observed in the research of Zawiślak et al. [16] and Jamshed et al. [67].

#### 3.2.3. Protein Oxidation

The oxidation of proteins has an adverse effect on the nutritional composition of meat products, so that the amount of essential amino acids such as histidine, tryptophan, cysteine, and methionine decrease due to the oxidation of proteins. The digestibility of proteins decreases, and toxic compounds are produced. This destructive process also has an adverse effect on the texture and color of food products containing proteins [68]. Researchers have found that the by-products of fat oxidation can simulate and increase the intensity of protein oxidation during the storage period, so the use of antioxidants is an effective solution to reduce the rate of fat and protein oxidation in meat products [69]. The protein oxidation in meat and meat products is evaluated by measuring the amount of carbonyl.

**Figure 5:**
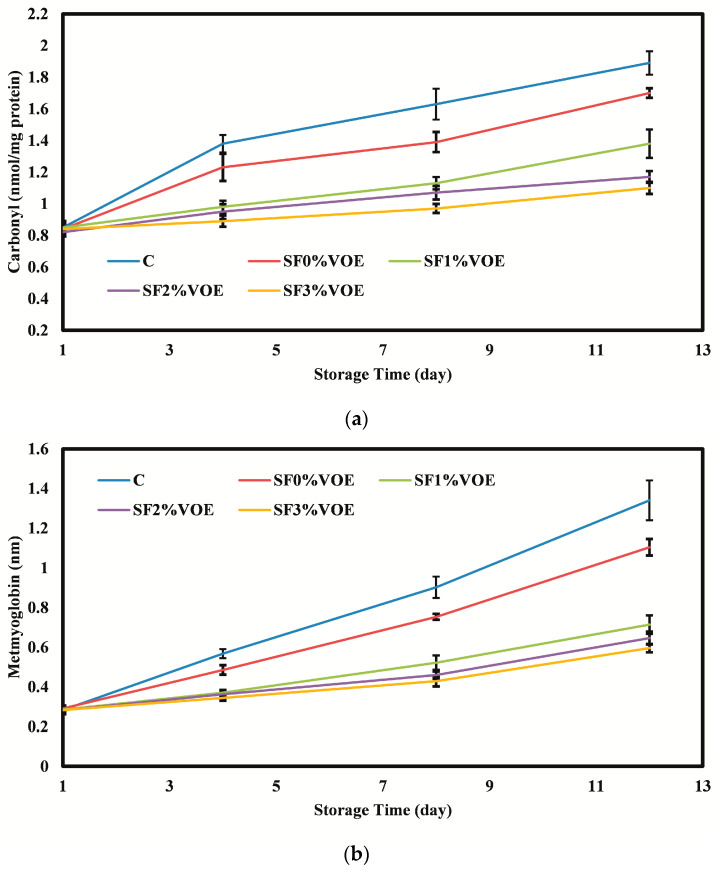
(**a**) Changes in the carbonyl and (**b**) metmyoglobin content of chicken fillets during the cold storage period. C: control sample; VOE: *Violata orodata* extract.

The results of the protein oxidation in chicken fillets (Figure 5a) showed that during the cold storage, due to the development of fat oxidation and the direct relationship between fat oxidation and protein oxidation, the carbonyl content of fillet samples increased significantly (*p* < 0.05), so that on the first day of storage, the carbonyl content of chicken fillets was in the range of 0.82–0.85 nmol/mg protein, and on the last day of storage, it reached the range of 1.10–1.89 nmol/mg protein. On different days of storage, the highest carbonyl content was for the control sample, and with an increase in the level of VOE in the active film, the amount of carbonyl production significantly decreased compared to the control (*p* < 0.05). Due to the remarkable antioxidant activity of the VOE, it was not far from expected to reduce the intensity of protein oxidation in the fillets packed with active films containing this extract. In line with the results of the present study, the effect of phenol-rich extracts in reducing protein oxidation in meat and meat products has been reported by other researchers [70].

#### 3.2.4. Metmyoglobin

The oxidation of meat pigments (myoglobin) and their transformation into metmyoglobin leads to a decrease in the acceptance of meat and meat products and affects the quality of these food products [71]. The results of the examination of metmyoglobin changes in packaged chicken fillets during the cold storage period are shown in Figure 5b. At first, the metmyoglobin content of fillet samples was in the range of 0.282–0.291 nm, and over time, the amount of this pigment in the meats increased significantly (*p* < 0.05), and the highest amounts of it was observed on the 12th day of storage (0.595–1.341 nm). Generally, the control sample had the highest amount of metmyoglobin, and the fillets packed in the active film containing the highest level of VOE (3%) had the lowest amount. Research has shown a direct relationship between fat oxidation and the production of metmyoglobin and the degradation of the color of meat and meat products. As a result of fat oxidation, free radicals are produced, which lead to the beginning of myoglobin oxidation (red color of meat) and its transformation into metmyoglobin (brown color pigment), a result of which the color of the meat during the storage period is degraded and undesirable. Therefore, using antioxidant agents can significantly reduce the intensity of metmyoglobin in meats by reducing the oxidation rate and producing fewer free radicals [72].

#### 3.2.5. Microbial Load

The results of the investigation of the microbial load of the chicken fillets are presented in Table 4 and show that, initially, the total viable bacteria count (TVBC), coliforms, and staph count of fillets were in the range of 2.65–2.77 log CFU/g, 2.14–2.19 log CFU/g, and 2.98–3.04 log CFU/g, respectively. The results of counting the microbial load of chicken fillets on the first day of storage indicated the high microbial quality and freshness of the chicken fillets on this day. During the cold storage period, due to the growth of microorganisms, the number of all bacteria studied in this research increased significantly (*p* < 0.05), and the highest number of them was observed on the last day of cold storage. The effect of the starch film without the extract in reducing the microbial load of the meat samples was significant compared to the control sample (*p* < 0.05), which is due to its gas barrier effect, so edible films and coatings prevent the exposure of the product to oxygen and thereby reduce the growth and proliferation rate of microorganisms. In this research, the number of different bacteria significantly decreased by adding the VOE and increasing its level from 1% to 3% in the starch films (*p* < 0.05). According to research, phenolic extracts cause bacteria cell death by destroying the cell wall, cytoplasmic membrane, and membrane proteins and interfering with the enzymes secreted from the membrane of microorganisms [73]. In previous studies, the significant antimicrobial activity of VOE has been confirmed [52,74]. In the research of Zarrabi et al. [74], cyclotides were introduced as one of the active VOE compounds with high antibacterial activity. The International Commission on Microbiological Specifications for Foods (ICMSF) has determined the maximum acceptable number for the total alive bacteria in meats to be 7 logs CFU/g [75], and the results of the present study demonstrated that the control sample and the meat packed in the starch film without the extract had a higher microbial load from the 8th day of cold storage; however, the meats packed in the starch films containing different levels of VOE were acceptable and safe until the last day of storage.

## 4. Conclusions

This research showed that adding different levels of VOE to starch films significantly improved the flexibility and gas and water vapor barrier characteristics of the active films. The active films containing VOE also indicated good light-blocking activity, especially against UV waves, and the films containing 2% and 3% VOE blocked the passage of UV light completely. As expected, there was a positive relationship between the concentration of VOE and the antibacterial and antioxidant activities of the films, and by increasing the VOE level in the films, the rate of changes in the physicochemical, oxidative, and microbiological parameters of the chicken fillets during the cold storage period significantly decreased, and by packing chicken fillets with active films containing 3% VOE, the microbial shelf life of fillets increased to at least eight days. In conclusion, these results demonstrated that it is possible to use active films based on potato starch containing VOE to improve the oxidative and microbiological quality of chicken fillets during the storage period in the refrigerator. Further investigation is required on the kinetics of VOE release from films, as well as on the methods for VOE stabilization.

## Figures and Tables

**Table 1:** Thickness, tensile strength (TS), Young’s modulus (YM), and elongation at the break point (EAB) of the films containing different levels of VOE.

Films	Thickness (mm)	TS (MPa)	YM (MPa)	EAB (%)
VOE0%	0.107 ± 0.003 a	39.84 ± 1.22 a	296.72 ± 1.54 a	11.09 ± 0.27 c
VOE1%	0.108 ± 0.006 a	38.52 ± 0.93 ab	293.19 ± 2.16 ab	11.44 ± 0.19 bc
VOE2%	0.109 ± 0.002 a	37.37 ± 1.04 bc	289.97 ± 2.10 bc	11.78 ± 0.22 b
VOE3%	0.111 ± 0.006 a	36.67 ± 0.88 c	286.44 ± 2.87 c	12.39 ± 0.08 a

Values represent the mean (*n* = 3) ± SD. Different letters in each column represent significant differences among samples at a 5% probability level. VOE: *Violata orodata* extract; TS: tensile strength; YM: Young’s modulus; EAB: elongation at the break point.

**Table 2:** Opacity and UV–visible transmission of active films containing different levels of VOE.

Films	Opacity (mm^−1^)	T280 (%)	T600 (%)
VOE0%	0.347 ± 0.006 d	44.61 ± 0.47 a	75.24 ± 0.72 a
VOE1%	0.491 ± 0.013 c	7.39 ± 0.31 b	34.19 ± 0.65 b
VOE2%	0.758 ± 0.019 b	0.00 ± 0.00 c	29.93 ± 0.89 c
VOE3%	1.014 ± 0.010 a	0.00 ± 0.00 c	17.87 ± 0.82 d

Values represent the mean (*n* = 3) ± SD. Different letters in each column represent significant differences among samples at a 5% probability level. VOE: *Violata orodata* extract.

**Table 3:** The bacterial growth inhibitory zones (mm) of active films containing different levels of VOE against food pathogen bacteria.

Films	*E. coli* (mm)	*S. aureus* (mm)	*S. typhimorium* (mm)
VOE0%	0.00 ± 0.00 d	0.00 ± 0.00 d	0.00 ± 0.00 d
VOE1%	11.56 ± 0.48 c	15.61 ± 0.30 c	14.00 ± 0.00 c
VOE2%	19.46 ± 0.83 b	24.67 ± 0.21 b	23.33 ± 0.48 b
VOE3%	29.96 ± 0.21 a	34.10 ± 0.44 a	31.50 ± 0.67 a

Values represent the mean (*n* = 3) ± SD. Different letters in each column represent significant differences among samples at a 5% probability level. VOE: *Violata orodata* extract.

**Table 4:** Comparison of the microbial load of chicken fillets during the 12-day cold storage period.

Samples	Storage Time (Day)	TAMB (Log CFU/g)	Coliforms (Log CFU/g)	Coagulase-Positive Staphylococci (Log CFU/g)
Control	1	2.75 ± 0.19 ^D,a^	2.16 ± 0.08 ^D,a^	3.02 ± 0.03 ^D,a^
	4	4.91 ± 0.05 ^C,a^	3.98 ± 0.07 ^C,a^	4.61 ± 0.09 ^C,a^
	8	7.49 ± 0.14 ^B,a^	5.13 ± 0.04 ^B,a^	5.99 ± 0.07 ^B,a^
	12	8.45 ± 0.11 ^A,a^	5.71 ± 0.11 ^A,a^	7.32 ± 0.04 ^A,a^
VOE0%	1	2.77 ± 0.08 ^D,a^	2.19 ± 0.05 ^D,a^	3.04 ± 0.08 ^D,a^
	4	4.32 ± 0.11 ^C,b^	3.33 ± 0.06 ^C,b^	4.00 ± 0.02 ^C,b^
	8	7.07 ± 0.07 ^B,b^	4.69 ± 0.04 ^B,b^	5.18 ± 0.03 ^B,b^
	12	7.42 ± 0.05 ^A,b^	5.27 ± 0.06 ^A,b^	6.25 ± 0.09 ^A,b^
VOE1%	1	2.70 ± 0.12 ^D,a^	2.17 ± 0.03 ^D,a^	3.00 ± 0.05 ^D,a^
	4	3.59 ± 0.07 ^C,c^	2.70 ± 0.09 ^C,c^	3.41 ± 0.03 ^C,c^
	8	4.56 ± 0.11 ^B,c^	3.35 ± 0.07 ^B,c^	3.90 ± 0.05 ^B,c^
	12	5.00 ± 0.17 ^A,c^	3.96 ± 0.08 ^A,c^	4.47 ± 0.02 ^A,c^
VOE2%	1	2.65 ± 0.09 ^D,a^	2.14 ± 0.08 ^D,a^	2.98 ± 0.04 ^D,a^
	4	3.28 ± 0.12 ^C,d^	2.48 ± 0.04 ^C,d^	3.24 ± 0.08 ^C,d^
	8	4.10 ± 0.03 ^B,d^	2.92 ± 0.03 ^B,d^	3.58 ± 0.04 ^B,d^
	12	4.67 ± 0.14 ^A,d^	3.41 ± 0.05 ^A,d^	3.88 ± 0.04 ^A,d^
VOE3%	1	2.66 ± 0.15 ^D,a^	2.14 ± 0.08 ^D,a^	2.98 ± 0.04 ^D,a^
	4	3.10 ± 0.04 ^C,e^	2.39 ± 0.02 ^C,e^	3.17 ± 0.02 ^C,d^
	8	3.81 ± 0.07 ^B,e^	2.80 ± 0.07 ^B,e^	3.44 ± 0.05 ^B,e^
	12	4.23 ± 0.09 ^A,e^	3.19 ± 0.04 ^A,e^	3.62 ± 0.08 ^A,e^

Values represent the mean (*n* = 3) ± SD. VOE: *Violata orodata* extract; TVBC: total viable bacteria count. a–e and A–D different letters indicate significant differences among samples and storage periods at a 5% probability level, respectively.

## Data Availability

The data that support the findings of this study are available within the manuscript.
